# The association between the number of symptoms and the severity of Post-COVID-Fatigue after SARS-CoV-2 infection treated in an outpatient setting

**DOI:** 10.1007/s00415-023-11752-9

**Published:** 2023-05-23

**Authors:** Lena Schmidbauer, Inge Kirchberger, Yvonne Goßlau, Tobias D. Warm, Alexander Hyhlik-Dürr, Jakob Linseisen, Christa Meisinger

**Affiliations:** 1 Institute for Medical Information Processing, Biometry, and Epidemiology (IBE), Ludwig-Maximilians-Universität München, Marchioninistraße 15, 81377 Munich, Germany; 2 Pettenkofer School of Public Health, Munich, Germany; 3 Epidemiology, Medical Faculty, University of Augsburg, Augsburg, Germany; 4 Vascular Surgery, Medical Faculty, University of Augsburg, Augsburg, Germany

**Keywords:** Post-COVID-Fatigue, FAS, ME/CFS, Outpatients

## Abstract

**Background:**

Post-COVID-Fatigue (PCF) is one of the most reported symptoms following SARS-CoV-2 infection. Currently, research on persistent symptoms focuses mainly on severe infections, while outpatients are rarely included in observations.

**Objective:**

To investigate whether the severity of PCF is related to the number of acute and persistent symptoms due to mild-to-moderate COVID-19 and to compare the most common symptoms during acute infection with the persistent symptoms in PCF patients.

**Methods:**

A total of 425 participants were examined after COVID-19 treated as an outpatient (median 249 days [IQR: 135; 322] after acute disease) at the site of University Hospital Augsburg, Germany. The Fatigue Assessment Scale (FAS) was used to quantify the severity of PCF. The number of symptoms (maximum 41) during acute infection and persistent symptoms (during the last 14 days before examination) were added up to sum scores. Multivariable linear regression models were used to show the association between the number of symptoms and PCF.

**Results:**

Of the 425 participants, 37% (*n* = 157) developed PCF; most were women (70%). The median number of symptoms was significantly higher in the PCF group than in the non-PCF group at both time points. In multivariable linear regression models, both sum scores were associated with PCF (acute symptoms: β-estimate per additional symptom [95%-CI]: 0.48 [0.39; 0.57], *p* < 0.0001); persistent symptoms: β-estimate per additional symptom [95%-CI]: 1.18 [1.02; 1.34], *p* < 0.0001). The acute symptoms strongest associated with PCF severity were difficulty concentrating, memory problems, dyspnea or shortness of breath on exertion, palpitations, and problems with movement coordination.

**Conclusion:**

Each additional symptom that occurs in COVID-19 increases the likelihood of suffering a higher severity of PCF. Further research is needed to identify the aetiology of PCF.

Trial registration: Nr. NCT04615026. Date of registration: November 4, 2020.

**Supplementary Information:**

The online version contains supplementary material available at 10.1007/s00415-023-11752-9.

## Introduction

Myalgic Encephalomyelitis/Chronic Fatigue Syndrome (ME/CFS) is a complex disease with profound dysregulation of the central nervous system and immune system, dysfunction of cellular energy metabolism, and ion transport and cardiovascular abnormalities [1–3]. Patients suffer persistent episodes of fatigue, cognitive dysfunction, depression, and other symptoms after minimal activity [1, 2, 4]. Depending on the severity, ME/CFS can have major consequences for work and daily functioning; about 30–60% of fatigue patients are unable to work [5, 6]. The aetiology of ME/CFS has not yet been fully researched, and the initial studies indicated women are more likely to be affected [7–11]. Likewise, this is a known (post-) complication of viral infections, such as Epstein–Barr virus, Q-fever, Ross-River virus, or giardiasis. The symptom persists for several months after infection. However, the severity of the infection does not determine the severity of the fatigue syndrome: mild viral infections can also lead to severe ME/CFS [7, 12, 13]. This has also been shown for SARS-CoV-2 infections: severe and mild disease courses can lead to ME/CFS [13–16]. Scientists at the Charité in Berlin called the symptom fatigue due to SARS-CoV-2 infection “Post-Covid-Fatigue” (PCF) [17]. During the pandemic, millions of people worldwide were and are infected with one of the different variants of the SARS-CoV-2 virus [18]. Of these, around 85% were or are being treated as outpatients [19], and most infected individuals with a mild-to-moderate severity of the infection also develop symptoms [20]. So far, the influential factors that increase the severity of PCF are largely unknown [18, 21]. In this context, it is not clear whether the number of symptoms has an impact on the severity of PCF.

Therefore, the primary objective of the present study was to investigate whether the number of symptoms during the acute SARS-CoV-2 infection and the number of persistent symptoms are associated with the severity of PCF in patients who were treated in an outpatient setting. A secondary objective was to compare the most common symptoms during acute infection with the most common persistent symptoms in PCF patients.

## Methods

The present study was based on data from the Corona Thrombosis Study (COVID-T), a single-center observational study carried out at the University Hospital of Augsburg, Germany.

The aim of the COVID-T study was to investigate the effects of a mild or moderate SARS-CoV-2 infection on the vascular system. Participants were recruited from local public health departments of the city of Augsburg and the county of Augsburg between October 10th, 2020, and November 6th, 2020. All individuals with a positive polymerase chain reaction (PCR) test, residing in the Augsburg area, at least 18 years old or older, were invited by the public health departments to participate in the study.

The examinations took place between November 2020 and May 2021 at the University Hospital of Augsburg, Germany. Due to the pandemic, additional precautions were necessary: the last positive smear had to be taken at least 14 days ago and the accompanying isolation measures had to be lifted.

**Fig. 1:**
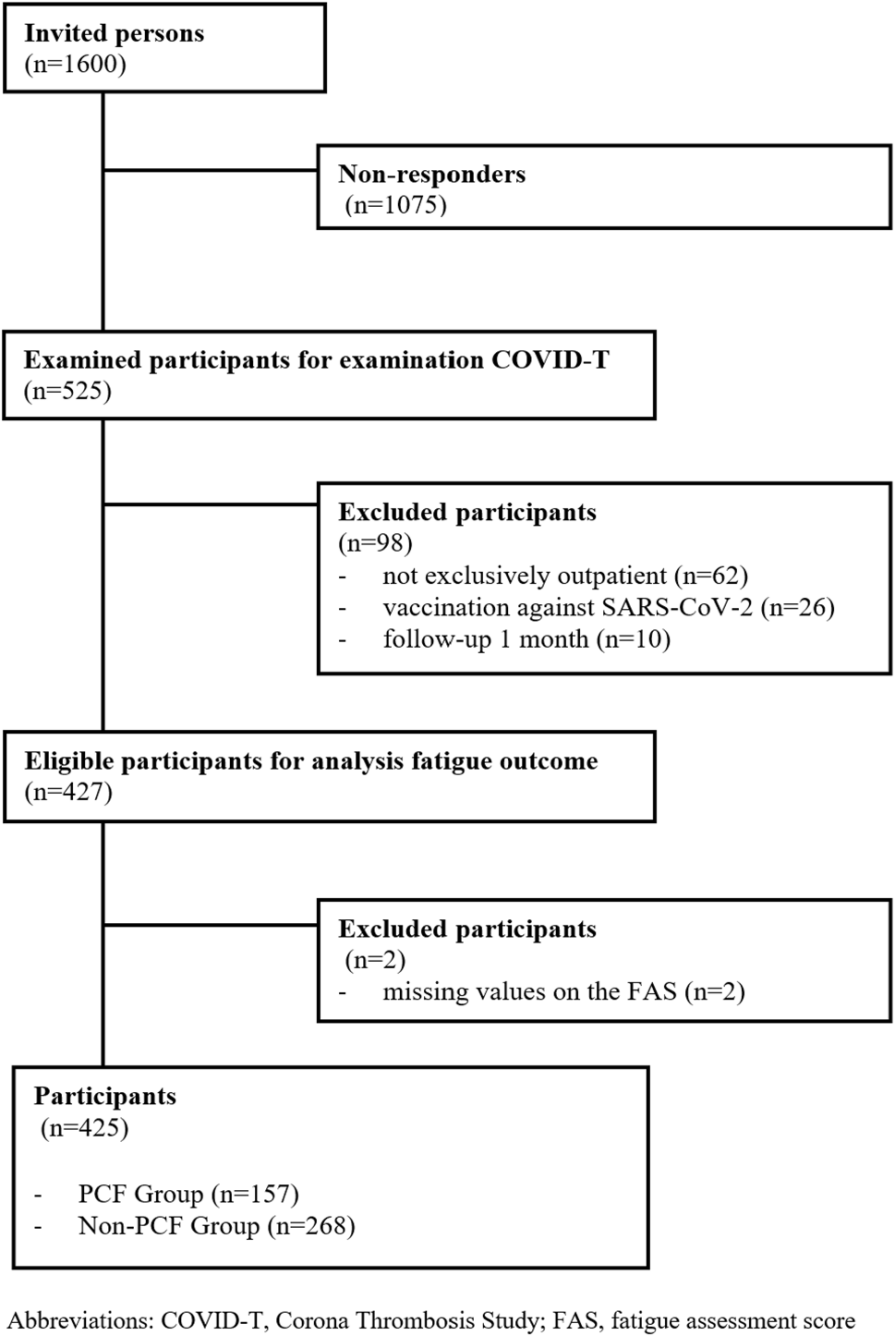
Study selection flowchart

Altogether, from the 1600 invited residents, 525 individuals could be examined; from these, 463 study participants were treated on an outpatient basis and thus included in the present study. For the analysis regarding PCF outcome, all individuals who were not treated as outpatients were excluded, as well as having had already received vaccination against SARS-CoV-2 infection and whose follow-up was less than 1 month after diagnosis. Additionally, participants with missing values on the Fatigue Assessment Scale (FAS) or other relevant variables were not included (*n* = 2), leaving 425 participants for the analysis (see Fig. 1).

The study was approved by the ethics committee of the Ludwig-Maximilians-Universität München (No. 20–735) and written informed consent was given by each study participant. The study was conducted in accordance with the Declaration of Helsinki.

### Data collection

The sociodemographic data of the participants were collected using a digital questionnaire via a tablet. In addition to the basic information about symptoms during the acute infection and persistent symptoms (during the last 14 days before examination), further information was also of interest to the study. Therefore, information about the overall medical history, smoking behavior, and subjective assessment of general physical and mental status was also requested. The following 42 symptoms or complaints were assessed: increased temperature (37.5–38.0 °C), fever (38.1° and above), chills, cold or runny nose, nasal congestion, sore throat or throat pain, dysphagia, cough, hemoptysis, dyspnea or shortness of breath at rest, dyspnea or shortness of breath on exertion, feeling of pressure or chest pain, palpitations, heartburn, nausea or vomiting, abdominal pain, diarrhea, flatulence, decreased appetite, muscle or joint pain, muscle weakness, muscle stiffness, problems coordinating movements, tingling arms or legs, visual impairment, epiphora, red eyes or conjunctivitis, cyanosis, parosmia, dysgeusia, headache, vertigo, fatigue or exhaustion, sleepiness, sleep disturbance, difficulties concentrating, memory impairment, depressed mood, anxiety or panic, mood swings, rash, and hair loss. Two new variables were created for every participant: one sum score of the symptoms during the acute infection and one sum score of the persistent symptoms. All symptoms, excluding the symptoms fatigue or exhaustion, were added up to form the sum scores.

### Fatigue assessment

The extent of PCF was measured using the FAS [22, 23] which is known to be a valid screening tool for fatigue in patients with chronic diseases. The FAS is a 10-item general fatigue questionnaire with five questions regarding physical fatigue and five questions about mental fatigue resulting in a total score of 50 (maximum score). The answers consist of the following options: *Never*, 5 score; *Sometimes*, 4 score; *Regularly*, 3 score; *Often*, 2 score; *Always*, 1 score. A score of < 22 indicates no fatigue, a score of 22–35 indicates moderate fatigue, and a score of > 35 indicates a high level of fatigue [22].

### Statistical methods

Descriptive statistics were either reported as mean with standard deviation (SD) or median with interquartile range (IQR) as appropriate. The differences of characteristics between the group of individuals with PCF (FAS score ≥ 22) compared to the non-PCF group (FAS score < 22) were tested using t test, Chi-square test, and Mann–Whitney *U* test as appropriate. Data were examined for normality using Quantile–Quantile plot (Q–Q plot).

Two multiple linear regression models were fitted to analyze the relationship with severity of PCF (FAS score as continuous variable) as outcome. The exposure-variables of interest were the symptom sum-scores (during acute infection; persistent symptoms). The covariates [age, sex, and body mass index (BMI)] added in the models were chosen based on the current literature. In addition, variables that indicated a significant difference between the PCF and non-PCF group in the descriptive analysis were included as confounders in the multivariable regression models. A formal test on interaction between sex and sum score was conducted, to examine for potential effect modification by sex. In addition, further analyses were performed. For each symptom, a group comparison was calculated using a Chi-square test. In succession, a multiple linear regression was then modeled for all significant symptoms, using the same covariates as in the model for the sum score. The median number of symptoms was calculated in all groups for both time periods. For the PCF group, the symptoms were contrasted to show a comparison of the most frequent symptoms at each time point.

In addition, further analyses were performed. For each symptom, a group comparison (PCF versus no PCF) was calculated using a Chi-square test. In succession, a multiple linear regression was then modeled for all significant symptoms, using the same covariates as in the model for the sum score. The median number of symptoms was calculated in all groups for both time periods. For the PCF group, the symptoms were contrasted to show a comparison of the most frequent symptoms at each time point.

All statistical analyses were carried out using SAS University Edition (version 5.2). The preselected statistical level of significance was considered *p* < 0.05.

## Results

### Participant characteristics

Of the 425 participants, 36.8% suffered from PCF at least 1 month after a mild-to-moderate infection with SARS-CoV-2; 30% (*n* = 128) had moderate fatigue and 6.8% (*n* = 29) had high level of fatigue. The age of study participants ranged between 19 and 87 years. Table 1 shows the sociodemographic characteristics and comorbidities according to participants with or without PCF. With 70%, the proportion of women among those affected with PCF was significantly higher than the proportion of men (30%). Individuals with lower education suffered significantly more often from PCF than participants with a higher education (*p* value = 0.0083). The remaining sociodemographic variables did not differ between the two groups.

**Table 1:** Baseline characteristics of the study participants by PCF (yes/no)

Characteristic	PCF *N* = 157	Non-PCF *N* = 267	*p* value*
Sex, *n/N* (%)			< 0.0001
Female	110/157 (70.06)	126/267 (47.19)	
Male	47/157 (29.94)	141/267 (52.81)	
Age (years), mean (SD)	47.08 (14.60)	47.11 (15.60)	0.9846
Marital status, *n/N* (%)			0.7145
Married	106/156 (67.95)	168/267 (62.92)	
Single	41/156 (26.28)	79/267 (29.59)	
Divorced	7/156 (4.49)	14/267 (5.24)	
Widowed	2/156 (1.28)	6/267 (2.25)	
Life partner, *n/N* (%)			0.09212
No	41/154 (26.62)	72/266 (27.07)	
Yes	113/154 (73.38)	194/266 (72.93)	
Highest school qualification, *n/N* (%)			0.0083
10 years of school or lower	92/157 (58.60)	121/267 (45.32)	
11 years of school or higher	65/157 (41.40)	146/267 (54.68)	
Smoking habit, *n/N* (%)			0.2245
Non-smoker	80/157 (50.96)	144/267 (53.93)	
Smoker	12/157 (7.64)	31/267 (11.61)	
Ex-smoker	65/157 (41.40)	92/267 (34.46)	
BMI (kg/m^2^), mean (SD)	26.14 (5.31)	25.75 (4.95)	0.4470
Time between examination date and first positive PCR test (days, median (IQR)	216 (116–323)	249 (135–322)	0.628
Comorbidities, *n/N* (%)			
Hypertension			0.3213
Yes	26/156 (16.67)	55/267 (20.60)	
No	130/156 (83.33)	212/267 (79.40)	
Coronary heart disease or angina pectoris			0.3746
Yes	8/156 (5.13)	9/267 (3.37)	
No	148/156 (94.87)	258/267 (96.63)	
Heart attack			0.3569
Yes	2/156 (1.28)	7/267 (2.62)	
No	154/156 (98.72)	260/267 (97.38)	
Diabetes			0.9582
Yes	6/156 (3.85)	10/267 (3.75)	
No	150/156 (96.15)	257/267 (96.25)	
Stroke			0.6459
Yes	2/156 (1.28)	5/267 (1.87)	
No	154/156 (98.72)	262/267 (98.13)	
Cancer			0.9173
Yes	8/157 (5.10)	13/267 (4.87)	
No	149/157 (94.90)	254/267 (95.13)	
Depression			0.0005
Yes	23/157 (14.65)	13/267 (4.87)	
No	134/157 (85.35)	254/267 (95.13)	
Anxiety disorder			0.0074
Yes	15/156 (9.62)	9/267 (3.37)	
No	141/156 (90.38)	258/267 (96.63)	
Chronic bronchitis			0.4899
Yes	10/155 (6.45)	13/267 (4.87)	
No	145/155 (93.55)	254/267 (95.13)	
Autoimmune disease			0.0476
Yes	17/156 (10.90)	15/267 (5.62)	
No	139/156 (89.10)	252/267 (94.38)	

BMI, body mass index; IQR, interquartile range; PCR, positive polymerase chain reaction; SD, standard deviation

*Calculated using *T* test, Mann–Whitney *U* test, or Chi-squared test

In both groups, the most common comorbidity was hypertension, with 17% in the PCF and 21% in the non-PCF group, but the proportions did not significantly differ between groups. However, depression, anxiety disorder, and autoimmune disease were more frequently found in PCF patients. For example, depression affected 15% of the participants in the PCF group and 5% in the non-PCF group (*p* value = 0.0005).

### Symptoms at the time of acute infection and PCF

Table 2 shows the multivariable linear regression model results for the association between the sum score of symptoms at the time of acute infection and PCF severity. The number of symptoms during the acute phase was significantly associated with the severity of PCF after adjustment for age, sex, BMI, highest school qualification, autoimmune disease, anxiety disorder, and depression (β-estimate per additional symptom [95%-CI]: 0.48 [0.39; 0.57], *p* < 0.0001).

**Table 2:** Multivariable linear regression model for the association between symptoms (sum score) at the time of acute infection and PCF severity

Variable	β-estimate	95% CI		*p* value
Sum-score acute phase	0.48	0.39	0.57	< 0.0001
Age	− 0.03	− 0.07	0.02	0.1988
Sex	1.64	0.32	2.96	0.0148
BMI	0.10	− 0.03	0.23	0.1161
Highest school qualification	− 0.64	− 1.29	− 0.00	0.0496
Autoimmune disease	0.46	− 1.95	2.87	0.7102
Anxiety disorder	3.70	0.81	6.59	0.0122
Depression	4.13	1.65	6.61	0.0012

BMI, body mass index; CI, confidence interval

As shown in Table 3, a statistically significant relationship between the number of persistent symptoms and severity of PCF existed. The β -estimate per additional persistent symptom [95%-CI] was 1.18 [1.02; 1.34].

**Table 3:** Multivariable linear regression model of the association between persistent symptoms and PCF severity

Variable	β-estimate	95% CI		*p* value
Sum-score persistent symptoms	1.18	1.02	1.34	< 0.0001
Age	− 0.05	− 0.09	− 0.01	0.0176
Sex	1.20	− 0.01	2.40	0.0516
BMI	0.06	− 0.06	0.18	0.3095
Highest school qualification	− 0.70	− 1.29	− 0.11	0.0195
Autoimmune disease	0.44	− 1.76	2.63	0.6948
Anxiety disorder	0.99	− 1.66	3.64	0.4626
Depression	1.69	− 0.63	4.00	0.1533

BMI, body mass index; CI, confidence interval

There was no significant interaction between sex and both sum scores of symptoms. Therefore, no sex-specific analyses were conducted. The model regarding symptoms during the acute infection explained 32% of the FAS score. The persistent symptoms model showed an adjusted *R*^2^ of 0.42.

### Associations between single symptoms and severity of PCF

In the group comparison between PCF and non-PCF during the acute phase, a significant difference was found in 33 out of 41 symptoms. The following eight symptoms were the only ones that did not show a significant change in the acute infection in the group comparison: fever, runny nose, nasal congestion, hemoptysis, heartburn, rash, increased temperature, and cough. The full list of comparisons between the PCF group and the non-PCF group in terms of the 41 possible symptoms of acute infection is presented in Table S1 and for persistent symptoms in Table S2 in the Supplement.

Linear regressions were calculated for each of the 33 symptoms during the acute infection using the same variables for adjustment as in the sum score model: age, sex, BMI, highest school grade, autoimmune disease, anxiety disorder, and depression. The results of the regression models and the adjusted R^2^ are reported in Table S3 in the supplement. Of the 33 multiple linear regression models of symptoms in the acute phase, 30 associations were significant. The symptoms with the strongest association with the severity of PCF were difficulty concentrating (β-estimate [95%-CI]: 5.18 [3.86; 6.50], *p* < 0.0001) *R*^2^ of 0.2636, memory problems (β-estimate [95%-CI]: 5.37 [3.79; 6.95], *p* < 0.0001) *R*^2^ of 0.2398, dyspnea or shortness of breath on exertion (β-estimate [95%-CI]: 4.35 [3.00; 5.69], *p* < 0.0001) *R*^2^ of 0.23 26, followed by palpitations (*R*^2^ of 0.2301) and problems with movement coordination (*R*^2^ of 0.2199). The three symptoms not significantly related to the severity of PCF according to our calculation were sore throat or throat pain, red eyes or conjunctivitis, and taste disturbance.

### Differences between symptoms during the acute infection and persistent symptoms

The median number of symptoms at the time of acute infection shows as 16 with a range of 2–33 in the PCF group and nine with a range of 0–30 in the non-PCF group. In terms of persistent symptoms, the median was five with a range of 0–24 in the PCF group and one with a range of 0–12 in the non-PCF group.

The full list of symptom frequencies and the differences regarding the 41 possible symptoms between both time points for the PCF group is presented in Table S4 in the supplement. The most common symptom at the time point of acute infection was headache with 80% (127/157), followed by sleepiness (125/157) and muscle or joint pain (123/157).

The largest difference of symptom frequency between the two time points was found for dyspnea or shortness breath on exertion, followed by decreased appetite. Overall, the number of reports was decreased for 28 symptoms, and the frequency of the symptom flatulence remained consistent, reported by 45 study participants at both time points. Three symptoms were more frequently reported during the persistent period than in the acute phase, including memory impairment, rash, and heartburn. The symptom hemoptysis has not been reported as persistent symptom.

## Discussion

The present study demonstrated that after a mild-to-moderate SARS-CoV-2 infection 37% of the participants developed PCF (FAS score ≥ 22) at least 1 month after the acute infection. The number of symptoms at the time point of acute infection and after a median of 249 days after infection (persistent symptoms) was independently associated with the severity of PCF.

The proportion of participants suffering from PCF after a mild-to-moderate SARS-CoV-2 infection in the present study is noteworthy. This is particularly important, because the PCF group met diagnostic criteria for fatigue, as their FAS score was similar to those of other ME/CFS cohorts [24]. Whether the presence of PCF in our study population is going to stabilize at this level will be shown by a follow-up survey of this sample in 2022.

With 70%, the proportion of women in the PCF group was significantly higher than the proportion of men. This distribution is consistent with findings in the other studies [6–8, 10, 11, 13, 16, 25]. According to Townsden et al., one reason for this could be that women experience depression and anxiety disorders more often [6, 16]. An association between ME/CFS and depression is known, but it is not yet known which condition occurs first [6, 26]. The pre-existing comorbidities depression and anxiety disorder were also significantly different in our study comparing subjects with and without PCF. Despite the inclusion of these two comorbidities in the multivariable regression models, both the number of acute and persistent symptoms were independently positively associated with PCF severity. This effect was also seen in other studies despite different assessment instruments for fatigue were used [16]. Although more women reported symptoms than men [27] and women had more often depression, anxiety disorders, and stress [28], contrary to other studies [16], a possible effect modification by sex could be ruled out in the present study.

In the PCF group, the median number of symptoms at both time points were significantly higher than in the group without PCF. In the PCF group, a median of five persistent symptoms was reported. A comparison with other studies is limited due to the heterogeneity of the symptoms and the number of symptoms recorded.

Many studies reported only the frequency and duration of symptoms, not the number of symptoms per infected person. Consistent with the present study, Davis et al. found that most patients were not fully recovered after seven months. The patients who had not recovered had a higher FAS score than the recovered individuals [29]. In the study by Hartung et al. [16], it was reported that 19% of patients suffered from clinically relevant fatigue on average 9 months after infection, compared to 8% of the comparison group without COVID-19 (*p* < 0.001). In the study by van Kessel et al. [30], it was observed that approximately 10–35% of COVID-19 patients who received outpatient treatment develop persistent symptoms. Further studies also reported that many patients do not regain their full health after SARS-CoV-2 infection [6, 16, 25, 30–32].

In most prior investigations, fatigue was the most common or second most common post-acute symptom, even 2 months after the SARS-CoV-2 infection [10, 15, 16, 29–33]. As mentioned before, a comparison of symptoms with other studies is difficult. Most prior studies have collected far less than 41 symptoms. Nevertheless, our findings are largely consistent with many studies [30, 31]. Most symptoms decrease after the acute infection [31, 34], a finding which could be confirmed in the present study. In the PCF group, the most frequently reported persistent symptoms were difficulties concentrating (55%), followed by memory impairment (49%) and muscle or joint pain (41%). These results were similar to those of other studies [13, 15, 25, 29, 35], including a study by Peter et al., where 30 symptoms were clustered. The most common clusters were fatigue (including: rapid physical exhaustion, and chronic fatigue), neurocognitive impairment (including: difficulty concentrating, memory impairment, and confusion), and chest symptoms (including: shortness of breath, chest pain, and wheezing). In addition to these most severe symptoms, the least severe persistent symptoms were also comparable. In both studies, typical infection symptoms, such as fever and chills, greatly decreased during the acute phase of infection [13]. In the group comparison of PCF and non-PCF, 33 symptoms showed significant differences. The typical cold symptoms were present in both groups and were not significant, such as runny nose, fever, or cough. Of all the aforementioned 33 symptoms, 30 symptoms were observed to be significantly related to the severity of PCF. The symptoms concentration difficulties and memory problems showed the strongest associations. These results are similar to the findings of the systematic review by Joli et al. [25]. In that systematic review, fatigue (by definition) was always present; anhedonia, brain fog, and difficulty concentrating (up to 81%); myalgia (up to 55%); depression/anxiety (up to 47%); insomnia, sleep problems (up to 33%); and dementia or memory loss (up to 32%) [25]. Our results are also comparable to those of a meta-analysis by Lopes-Leon et al. including 15 studies from France, Italy, USA, Australia, UK, Mexico, Ireland, China and Egypt. Here, 55 persistent symptoms related to COVID-19 were studied. The five most common manifestations were fatigue, headache, attention deficit disorder, hair loss, and dyspnea. Headache was the most commonly reported symptom during acute infection in our study, and subjects continued to report it as a persistent symptom, placing them in the top third of persistent symptoms. The outcome attention deficit disorder from the meta-analysis is comparable to the symptom difficulty concentrating, which was the most frequently reported persistent symptom in the present study. Hair loss and dyspnea were listed in our study, but both outcomes were not as common as reported in the meta-analysis [15].

### Limitations and strengths

The first limitation in this study lies within the selected population, our analysis was based on as only adults from the Augsburg area were included. Ethnicity was not recorded, but based on the region, it can be assumed that most patients had German nationality. A transfer of the results to children as well as to other regions and ethnicities is therefore only possible to a limited extent. Second, only individuals with a mild-to-moderate COVID-19 course were included. Thus, the results are not transferable to severe and very severe infections. Third, we determined PCF using only the FAS score; subjects did not receive an official diagnosis of fatigue from a medical professional. Different validated questionnaires are used worldwide to determine fatigue. Therefore, comparability of results between studies is often difficult. Fourth, an important characteristic of ME/CFS is that the symptoms must persist for at least 6 months. Some respondents did not meet this criterion. The assignment of individuals to the PCF group may therefore be overestimated. Fifth, the retrospective nature of the study exposes the possibility of recall bias, which could impact the reliability of symptom prevalence estimates. Sixth, some symptoms may have arisen independently of the SARS-CoV-2 infection and were incorrectly added. Seventh, there are symptoms that overlap with other symptoms. Therefore, it cannot be excluded that some symptoms were overestimated, and others underestimated. In addition, some symptoms may have disappeared and reappeared in the interim or may have appeared weeks or months after the initial infection.

Strong points in this study are: first, the analysis was based on a homogeneous sample, since only outpatients were included. With 425 participants, the sample was relatively large compared to other study populations of outpatients. Second, only individuals who had a positive PCR test, and thus, a confirmed SARS-CoV-2 infection were included and contacted by the public health departments of the Augsburg region. Third, the median days between initial infection and interview were 249 days, and thus, the initial infection occurred more than half a year earlier; therefore, the comparison with ME/CFS patients might be defensible. Fourth, the number of symptoms collected is very high compared to the literature.

## Conclusion

With millions of people infected with COVID-19, the number of those suffering from PCF is rapidly increasing. This cross-sectional observational study found that the number of symptoms is associated with the severity of PCF. COVID-19 patients with many symptoms should be observed by medical professionals with regard to the manifestation of PCF. It is important that the awareness of PCF and a subsequent clinical diagnosis of ME/CFS increase, as this disease is a burden on individual patients and their families, as well as on ambulatory care, public health, and the economy. Further studies are necessary to uncover the aetiology of PCF and to find appropriate and well-timed preventive and therapeutic approaches for patients.

## Supplementary Information

Below is the link to the electronic supplementary material.

Supplementary file1 (DOCX 53 kb)

## Data Availability

The dataset generated during and/or analyzed during the current study is not publicly available due to data protection aspects but are available in an anonymized form from the corresponding author on reasonable request.
