# Supplementary material for: The association between the number of symptoms and the severity of Post-COVID-Fatigue after SARS-CoV-2 infection treated in an outpatient setting
Source: J Neurol. 2023 May 23;270(7):3294–302. doi: 10.1007/s00415-023-11752-9 (PMC10204671; doi:10.1007/s00415-023-11752-9)
Supplement: Supplementary file 1 — Supplementary file1 (DOCX 53 kb) [file 415_2023_11752_MOESM1_ESM.docx]

Appendices

**Supplemental materials**

Table S1: Group comparison of 41 acute symptoms between PCF group and Non-PCF group

| Symptoms | PCF  N=157 | Non-PCF  N=267 | p- value |
| --- | --- | --- | --- |
| Increased temperature  Yes  No | 89/156  67/156 | 140/267  127/267 | 0.4132 |
| Fever  Yes  No | 75/156  81/156 | 110/267  157/267 | 0.2025 |
| Chills  Yes  No | 87/157  70/157 | 85/267  182/267 | 2.979e-06 |
| Cold or runny nose  Yes  No | 86/155  69/155 | 119/267  148/267 | 0.03925 |
| Nasal congestion  Yes  No | 76/156  80/156 | 100/267  167/267 | 0.03034 |
| Sore throat or throat pain  Yes  No | 52/157  105/157 | 121/267  146/267 | 2.714e-05 |
| Pain when swallowing, cough  Yes  No | 59/156  97/156 | 57/267  210/267 | 0.0003839 |
| Cough  Yes  No | 99/157  58/157 | 148/267  119/267 | 0.1511 |
| Haemoptysis  Yes  No | 3/156  153/156 | 2/267  265/267 | 0.5407 |
| Dyspnea or shortness of breath at rest  Yes  No | 76/156  80/156 | 56/267  76/267 | 5.439e-09 |
| Feeling of pressure or chest pain  Yes  No | 82/156  74/156 | 72/267  195/267 | 2.287e-07 |
| Palpitations  Yes  No | 71/155  84/155 | 50/267  217/267 | 5.946e-09 |
| Heartburn  Yes  No | 20/155  135/155 | 23/267  244/267 | 0.216 |
| Nausea or vomiting  Yes  No | 33/157  124/157 | 25/267  241/267 | 0.001325 |
| Abdominal pain  Yes  No | 31/156  125/156 | 22/267  245/267 | 0.0008547 |
| Diarrhea  Yes  No | 66/157  91/157 | 62/267  205/267 | 7.306e-05 |
| Flatulence  Yes  No | 45/156  111/156 | 28/267  239/267 | 2.762e-06 |
| Loss of appetite  Yes  No | 109/156  47/156 | 126/267  141/267 | 9.515e-06 |
| Muscle or joint pain  Yes  No | 122/156  34/156 | 159/267  108/267 | 0.0001372 |
| Muscle weakness  Yes  No | 72/156  84/156 | 72/267  195/267 | 9.161e-05 |
| Muscle stiffness  Yes  No | 35/156  121/156 | 20/267  247/267 | 2.047e-05 |
| Problems coordinating movements  Yes  No | 40/157  117/157 | 18/267  249/267 | 1.327e-07 |
| Feeling of pinpricks in the arms and legs  Yes  No | 27/156  129/156 | 25/267  242/267 | 0.02462 |
| Visual impairment  Yes  No | 21/156  135/156 | 15/267  252/267 | 0.009088 |
| Tearing eyes  Yes  No | 37/156  119/156 | 30/267  237/267 | 0.001136 |
| Red eyes or conjunctivitis  Yes  No | 19/156  137/156 | 16/267  251/267 | 0.04079 |
| Cyanosis  Yes  No | 7/156  149/156 | 1/267  266/267 | 0.008637 |
| Disturbance of sense of smell  Yes  No | 7/156  149/156 | 1/267  266/267 | 0.000104 |
| Disturbance of sense of taste  Yes  No | 113/156  44/156 | 155/267  212/267 | 0.00567 |
| Headache  Yes  No | 126/157  31/157 | 154/267  113/267 | 3.587e-06 |
| Vertigo  Yes  No | 87/157  69/157 | 63/267  204/267 | 5.089e-11 |
| Sleepiness  Yes  No | 125/157  32/157 | 157/267  110/267 | 1.877e-05_ |
| Sleep disorder  Yes  No | 69/156  87/156 | 55/267  212/267 | 4.636e-07 |
| Difficulties concentrating  Yes  No | 104/157  53/157 | 83/267  184/267 | 3.948e-12 |
| Memory impairment  Yes  No | 72/156  85/156 | 34/267  233/267 | 6.865e-14 |
| Depressed mood  Yes  No | 25/156  131/156 | 12/267  255/267 | 6.024e-10 |
| Anxiety or panic  Yes  No | 55/156  101/156 | 36/267  231/267 | 2.816e-07 |
| Mood swings  Yes  No | 72/157  85/157 | 50/267  217/267 | 4.964e-09 |
| Rash  Yes  No | 11/156  145/156 | 17/267  250/267 | 0.9439 |
| Hair loss  Yes  No | 31/156  125/156 | 17/267  250/267 | 4.778e-05 |

Table S2: Group comparison of 41 persistent symptoms between PCF group and Non-PCF group

| Symptoms | PCF  N=157 | Non-PCF  N=267 | p- value |
| --- | --- | --- | --- |
| Increased temperature  Yes  No | 5/156  151/156 | 0/267  267/267 | 0.01326 |
| Fever  Yes  No | 2/156  154/156 | 0/267  267/267 | 0.2627 |
| Chills  Yes  No | 5/156  151/156 | 1/267  266/267 | 0.05127 |
| Cold or runny nose  Yes  No | 30/155  125/155 | 36/267  231/267 | 0.1438 |
| Nasal congestion  Yes  No | 27/156  129/156 | 37/267  230/267 | 0.4152 |
| Sore throat or throat pain  Yes  No | 13/155  142/155 | 8/267  259/267 | 0.02623 |
| Pain when swallowing, cough  Yes  No | 5/156  151/156 | 4/267  262/267 | 0.4129 |
| Cough  Yes  No | 23/156  133/156 | 19/267  248/267 | 0.01815 |
| Haemoptysis  Yes  No | 0/156  156/156 | 0/267  266/267 | - |
| Dyspnea or shortness of breath at rest  Yes  No | 21/156  135/156 | 8/267  259/267 | 9.225e-05 |
| Dyspnea or shortness of breath on exertion  Yes  No | 63/156  93/156 | 39/267  228/267 | 4.575e-09 |
| Feeling of pressure or chest pain  Yes  No | 29/155  126/155 | 14/267  253/267 | 2.221e-05 |
| Palpitations  Yes  No | 35/155  120/155 | 21/267  246/267 | 3.373e-05 |
| Heartburn  Yes  No | 21/155  134/155 | 37/267  230/267 | 1 |
| Nausea or vomiting  Yes  No | 9/156  147/156 | 3/267  264/267 | 0.01339 |
| Abdominal pain  Yes  No | 21/156  135/156 | 6/266  260/266 | 1.46e-05 |
| Diarrhea  Yes  No | 19/156  137/156 | 9/267  258/267 | 0.0009226 |
| Flatulence  Yes  No | 45/155  111/155 | 30/267  237/267 | 8.851e-06 |
| Loss of appetite  Yes  No | 13/155  142/155 | 2/267  265/267 | 0.0001375 |
| Muscle or joint pain  Yes  No | 64/156  93/156 | 28/267  239/267 | 6.883e-13 |
| Muscle weakness  Yes  No | 26/156  130/156 | 8/267  259/267 | 1.554e-06 |
| Muscle stiffness  Yes  No | 23/156  133/156 | 8/266  258/266 | 1.976e-05 |
| Problems coordinating movements  Yes  No | 15/156  141/156 | 3/265  262/265 | 9.388e-05 |
| Feeling of pinpricks in the arms and legs  Yes  No | 13/156  143/156 | 11/267  256/267 | 0.1119 |
| Visual impairment  Yes  No | 14/156  142/156 | 5/267  262/267 | 0.001583 |
| Tearing eyes  Yes  No | 20/156  136/156 | 16/267  251/267 | 0.0246 |
| Red eyes or conjunctivitis  Yes  No | 9/156  147/156 | 6/267  261/267 | 0.1058 |
| Cyanosis  Yes  No | 3/156  153/156 | 2/267  265/267 | 0.5407 |
| Disturbance of sense of taste  Yes  No | 33/157  122/157 | 35/267  232/267 | 0.03879 |
| Headache  Yes  No | 58/156  98/156 | 44/267  223/267 | 2.813e-06 |
| Vertigo  Yes  No | 25/156  131/156 | 12/267  255/267 | 0.000108 |
| Sleepiness  Yes  No | 64/157  93/157 | 18/267  93/267 | 2.2e-16 |
| Sleep disorder  Yes  No | 56/156  100/156 | 42/267  225/267 | 3.767e-06 |
| Difficulties concentrating  Yes  No | 86/156  70/156 | 20/267  246/267 | 2.2e-16 |
| Memory impairment  Yes  No | 77/156  79/156 | 15/267  252/267 | 2.2e-16 |
| Depressed mood  Yes  No | 50/156  106/156 | 19/267  248/267 | 5.362e-11 |
| Anxiety or panic  Yes  No | 21/156  135/156 | 10/267  257/267 | 0.0004544 |
| Mood swings  Yes  No | 56/156  100/156 | 30/267  237/267 | 2.595e-09 |
| Rash  Yes  No | 14/156  142/156 | 11/267  255/267 | 0.06891 |
| Hair loss  Yes  No | 24/156  132/156 | 16/267  250/267 | 0.002702 |

Table S3 shows the results of the linear regressions for the association of symptoms at the time of acute infection and severity of PCF. All symptoms which showed significant differences in the group comparisons were included. The adjustment variables for the regression models of each symptom were: age, sex, BMI, highest grade in school, autoimmune disease, anxiety disorder, depression.

| Table S3: Results of the linear regressions for the association between symptoms at the time of acute infection and PCF severityAcute Symptom | β -estimate | 95% CI | | p-value | R2 | |
| --- | --- | --- | --- | --- | --- | --- |
| Chills | 14.44 | 10.18 | 18.70 | <0.001 | 0.1985 | |
| Sore throat or throat pain | 3.27 | 1.92 | 4.61 | 4.767 | 0.2016 | |
| Pain when swallowing | 3.16 | 2.10 | 5.13 | <0.001 | 0.2003 | |
| Dyspnea or shortness of breath at rest | 4.11 | 2.66 | 5.55 | <0.001 | 0.2167 | |
| Dyspnea or shortness of breath on exertion | 4.35 | 3.00 | 5.69 | <0.001 | 0.2326 | |
| Feeling of pressure or chest pain | 3.38 | 1.96 | 4.79 | <0.001 | 0.2001 | |
| Palpitations | 4.73 | 3.23 | 6.23 | <0.001 | 0.2301 | |
| Nausea or vomiting | 2.32 | 0.29 | 4.35 | 0.025 | 0.1671 | |
| Abdominal pain | 4.96 | 2.91 | 7.00 | <0.001 | 0.2016 | |
| Diarrhea | 1.84 | 0.32 | 3.35 | 0.018 | 0.1691 | |
| Flatulence | 4.16 | 2.35 | 5.98 | <0.001 | 0.1972 | |
| Loss of appetite | 2.52 | 1.15 | 3.90 | <0.001 | 0.1835 | |
| Muscle or joint pain | 2.70 | 1.27 | 4.13 | <0.001 | 0.1848 | |
| Muscle weakness | 3.37 | 1.94 | 4.80 | <0.001 | 0.2003 | |
| Muscle stiffness | 4.62 | 2.58 | 6.66 | <0.001 | 0.1963 | |
| Problems coordinating movements | 6.07 | 3.99 | 8.14 | <0.001 | 0.2199 | |
| Feeling of pinpricks in the arms and legs | 4.55 | 2.51 | 6.60 | <0.001 | 0.195 | |
| Visual impairment | 2.97 | 0.47 | 5.47 | 0.020 | 0.1687 | |
| Tearing eyes | 3.39 | 1.51 | 5.26 | <0.001 | 0.1827 | |
| Red eyes or conjunctivitis | 1.56 | -0.97 | 4.10 | 0.226 | 0.1608 | |
| Cyanosis | 4.64 | -0.42 | 9.71 | 0.072 | 0.1643 | |
| Disturbance of sense of smell | 1.95 | 0.50 | 3.40 | 0.009 | 0.1717 | |
| Disturbance of sense of taste | 0.93 | -0.52 | 2.38 | 0.207 | 0.161 | |
| Headache | 4.05 | 2.60 | 5.50 | <0.001 | 0.2151 | |
| Vertigo | 3.54 | 2.10 | 4.97 | <0.001 | 0.203 | |
| Sleepiness | 3.50 | 2.06 | 4.94 | <0.001 | 0.2016 | |
| Sleep disorder | 4.24 | 2.76 | 5.73 | <0.001 | 0.2174 | |
| Difficulties concentrating | 5.18 | 3.86 | 6.50 | <0.001 | 0.2636 | |
| Memory impairment | 5.37 | 3.79 | 6.95 | <0.001 | 0.2398 | |
| Depressed mood | 4.11 | 2.52 | 5.70 | <0.001 | 0.207 | |
| Anxiety or panic | 3.95 | 2.26 | 5.63 | <0.001 | 0.1988 | |
| Mood swings | 3.58 | 2.03 | 5.13 | <0.001 | 0.1979 | |
| Rash | 2.31 | -0.45 | 5.07 | 0.101 | 0.1632 | |
| Hair loss | 4.29 | 2.07 | 6.50 | <0.001 | 0.1862 | |
| The adjustment variables were for each model: age, sex, BMI, highest grade of school, autoimmune disease, anxiety disorder, depression | | | | | |  |

Table S4 presents the 41 symptoms of the PCF group. They are ranked in descending order according to the frequency of acute infection. In column four, *Difference between the two time points*, the symptoms that were reported less over time are highlighted in green, and the symptoms that increased are highlighted in red; symptoms that remained constant are highlighted in orange.

Table S4: 41 symptoms at the time of acute infection and persistent symptoms in participants with PCF

| Symptom | Frequency at acute infection | Frequency of persistent symptoms | Difference between the two time points^*^ |
| --- | --- | --- | --- |
| Headache | 127 | 59 | 68 |
| Sleepiness | 125 | 64 | 61 |
| Muscel or joint pain | 123 | 65 | 58 |
| Dyspnea or shortness of breath on exertion | 120 | 21 | 99 |
| Parosmie | 118 | 42 | 76 |
| Dysgeusie | 113 | 33 | 80 |
| Decreased appetite | 109 | 13 | 96 |
| Difficulties concentration | 105 | 87 | 18 |
| Sore throat or throat pain | 105 | 13 | 92 |
| Cough | 99 | 23 | 76 |
| Increased temperature | 89 | 5 | 84 |
| Vertigo | 87 | 25 | 62 |
| Chills | 87 | 5 | 82 |
| Cold or runny nose | 86 | 30 | 56 |
| Feeling of pressure or chest pain | 82 | 29 | 53 |
| Nasal congestion | 76 | 27 | 49 |
| Dyspnea or shortness of breath at rest | 76 | 21 | 55 |
| Fever | 75 | 2 | 73 |
| Memory impairment | 72 | 77 | -5 |
| Mood swings | 72 | 56 | 16 |
| Muscle weakness | 72 | 26 | 46 |
| Palpitations | 71 | 35 | 36 |
| Depressed mood | 70 | 50 | 20 |
| Sleep disturbance | 69 | 56 | 13 |
| Diarrhoea | 66 | 19 | 47 |
| Dysphagia | 59 | 5 | 54 |
| Anxiety or panic | 55 | 21 | 34 |
| Flatulence | 45 | 45 | 0 |
| Problems coordinating movements | 40 | 15 | 25 |
| Epiphora | 37 | 20 | 17 |
| Muscle stiffness | 35 | 23 | 12 |
| Nausea or vomiting | 33 | 9 | 24 |
| Hair loss | 31 | 24 | 7 |
| Abdominal pain | 31 | 21 | 10 |
| Tingling arms or legs | 27 | 13 | 14 |
| Visual impairment | 22 | 15 | 7 |
| Heartburn | 20 | 21 | -1 |
| Red eyes or conjuncitivitis | 19 | 9 | 10 |
| Rash | 11 | 14 | -3 |
| Cyanosis | 7 | 3 | 4 |
| Haemoptysis | 3 | 0 | 3 |
| *Green highlighted fields indicate that the symptom decreased in frequency over time; orange highlighted fields indicate that the symptom is consistent over time; red highlighted fields indicate that the symptom increased in frequency over time. | | | |
